# Wake up, College of Nursing

**Published:** 1919-01-04

**Authors:** C. Seymour Yapp

**Affiliations:** Matron, Lake Hospital, Ashton-Under-Lyne.


*To the Editor of* The Hospital.

### WAKE UP, COLLEGE OF NURSING.

### Some Signs of Awakening.

### For and Against "Ierne's" Proposals.

*To the Editor of* The Hospital.

Sir,—Most nurses will agree with the remarks of "Ierne" in The Hospital of December 28 as to the profoundly unsatisfactory economic conditions under which many nurses work to-day. But it is doubtful whether the College of Nursing should be called upon to deal with these particular difficulties. The College has consistently adhered to its programme since its inception on February 23, 1916. It set out to (1) organise our profession. To-day it has nearly 11,000 members. Has any existing Nurses' Association that number of fully-trained members or anything approaching it? (2) Secure State Registration. The Registration Bill is in preparation. It is impossible to draw up such a Bill hurriedly; criticism and revision are inevitable, but the Bill will certainly be presented as soon as possible. (3) Establish a uniform curriculum of training. This is proceeding, but with hospital staffs so depleted of sisters and doctors the present is not an auspicious time to launch it. (4) Establish lectureships and scholarships. The scholarships are already in existence. According to your correspondents, there exists a feeling that there is no immediate material advantage to be gained by joining the College. A candidate must satisfy the Council of the College that she has received adequate training as a nurse, in recognition of which she is granted its certificate. Well, even to-day this has quite a definite commercial value, although nothing to be compared with what it will command in the future. The value of membership of the College cannot be expressed in terms of £ s. d. I take it that the College is primarily concerned with the academic, and not with the economic, side of the nurse's career, although they are dependent one upon the other. The former up till now has had no recognised status; this once established, material recognition follows. But certainly the economic side is of paramount importance to trained nurses to-day; adequate remuneration and marked improvement in working conditions in every branch of our profession demand our serious attention, and we should organise immediately to that end. Is there any objection to the formation of an association, composed of members of the College, to deal with the economic side of the profession, under the auspices of the College Council? I under-

**The Editor's Letter-Box**—(*continued*).

stand that the College has the question of salaries and "off-duty time" under consideration, but I feel that as a College it should concern itself mainly with the academic side of the profession, and that the economic side should be dealt with by another body affiliated to, and under the auspices of, the College. Anything in the nature of Trade Unionism would be quite against nursing traditions, but there are ways and means of securing adequate recompense for skilled service for a well-organised body of workers. With legislation, and even without it, as the College becomes more powerful the economic side will automatically improve, because all Government branches of the profession and all creditable nursing homes will make it a *sine qua non* that the nurses they employ shall be members of the College, and, of course, we shall fix our own minimum rate of remuneration. In order to fulfil the requirements of any modern, comprehensive curriculum, nurses will have to be given reasonable facilities for study, attendance at lectures at reasonable hours—not when they are tired out after a day's hard work; and this will bring about better conditions of training. Hospitals not granting these facilities would not be recognised by the College, and very shortly, as a result, they would have no probationers to train. So eventually the College will, to an appreciable extent, control the economic nursing conditions in hospitals.

We must gratefully admit that the College has accomplished wonderful things, working under great difficulties, especially when we consider how indifferent many nurses are to their own interests. Mistakes it will probably make—and unmake; all live organisations do; but as we have the power to elect whom we will to manage our affairs for us, we can ensure that the Council of the College does not go on making them for twenty-five years! Nurses have it absolutely in their own hands to make their profession what they will. The greater the number who join the College, the more powerful will it be to secure legislation to protect their interests.

C. Seymour Yapp,
Matron, Lake Hospital, Ashton-under-Lyne.

